# Induced Myopia Secondary to Blunt Trauma

**DOI:** 10.1155/2019/1632828

**Published:** 2019-12-30

**Authors:** Mohammad Reza Sedaghat, Hamed Momeni-Moghaddam, Shehzad A. Naroo, Mohammad Etezad-Razavi, Majid Moshirfar

**Affiliations:** ^1^Eye Research Center, Mashhad University of Medical Sciences, Mashhad, Iran; ^2^Health Promotion Research Center, Zahedan University of Medical Sciences, Zahedan, Iran; ^3^Department of Optometry, School of Paramedical Sciences, Mashhad University of Medical Sciences, Mashhad, Iran; ^4^Aston University, Ophthalmic Research Group, School of Life and Health Sciences, Aston Triangle, Birmingham B4 7ET, UK; ^5^John A. Moran Eye Center, Department of Ophthalmology and Visual Sciences, School of Medicine, University of Utah, USA; ^6^Utah Lions Eye Bank, Murray, UT, USA; ^7^HDR Research Center, Hoopes Vision, 11820 S. State Street Suite #200, Draper, UT 84020, USA

## Abstract

A 28-year-old young man presented with sudden and severe visual loss in the left eye, starting three days ago following blunt head trauma from a closed fist. Vision was not improved to better than 0.4 in the left eye. Slit lamp examinations revealed asymmetric anterior chamber depth (ACD) with shallow ACD in the left eye. The key learning in this report is the use of anterior segment optical coherence tomography (AS-OCT) to better document the anterior segment changes following the blunt trauma. AS-OCT was used to visualize the forward displacement of the iris-crystalline lens diaphragm secondary to ciliary body anterior rotation and ciliochoroidal effusion. There was a temporary myopic shift about 7.00 D which resolved after 15 days.

## 1. Introduction

Traumatic myopia is a clinical entity that may be seen following ocular blunt trauma and is characterized with a usual range of -1.00 to -6.00 diopters (D) in the injured eye, or occasionally in both eyes [1]. It is sudden onset and usually transient, recovering within a few weeks after the trauma, although some cases may be long-standing [2]. Possible etiologic causes for this condition are as follows: spasm of the ciliary body, increased crystalline lens effective power secondary to its forward shift, ciliochoroidal effusion causing forward displacement of the crystalline lens-iris diaphragm, axial thickening of the natural lens, and other sources of choroidals which could undoubtedly produce the same refractive issues such as varicella infection [2–4]. Although the closed head trauma may produce decreased visual acuity secondary to the bilateral spasm of accommodation, one case was reported with persistent myopia which was still present three months after trauma. Interestingly, there was no significant change in the anterior chamber depth between the two eyes (0.09 mm) and nearly absolute elimination of myopia following a cycloplegic refraction [5]. This report details the initial presentation and the outcome after two weeks of a case with traumatic myopia in one eye following the blunt head trauma from a closed fist. Although there are several reports of traumatic myopia with previously mentioned etiologies in the literature [1–3, 6–9], the key learning of this report is the use of anterior segment optical coherence tomography (AS-OCT) to better detail the anterior segment changes following the blunt trauma.

## 2. Case Presentation

A 28-year-old man presented with sudden and severe reduced vision in the left eye, following the blunt head trauma three days ago from a closed fist. Uncorrected distance visual acuity recorded as decimal notation was 0.9^−^ in the right eye and 0.1 in the left eye, respectively. The best-corrected distance visual acuity was 1.0 in the right eye with a refraction −0.75/−0.75 × 94° and 0.4 in the left eye with a refraction −5.50/−1.00 × 108°.

**Figure 1:**
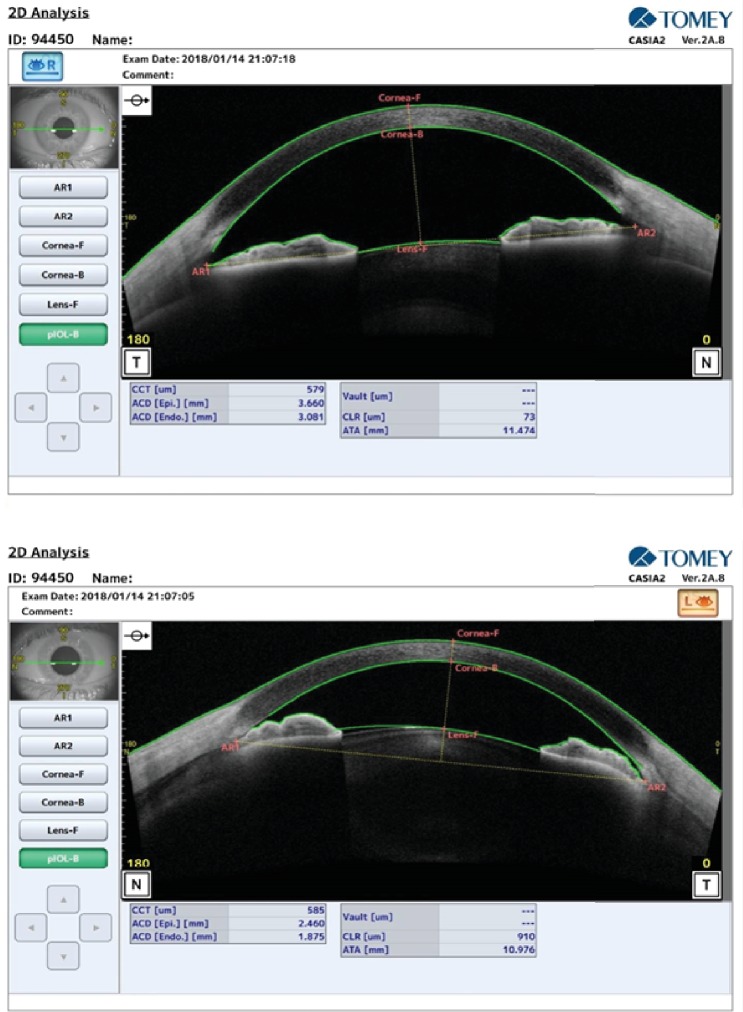
Anterior segment OCT three days after the trauma in the right and left eyes.

Slit lamp examinations revealed a normal cornea and clear crystalline lens. The only abnormal finding noted was asymmetric anterior chamber depth (ACD) with shallow ACD in the left eye. This was confirmed using the CASIA2 anterior segment optical coherence tomography (Tomey Corporation, Nagoya, Japan) (Figure 1).

Assessment of pupillary reflexes showed no relative afferent pupillary defect. Intraocular pressure was 13.6 mmHg in the right eye and 7.9 mmHg in the left eye using a Topcon noncontact tonometer (Topcon Corporation, Tokyo, Japan). Dilated fundus examination showed a normal retina, macula, and optic disc in both eyes. Keratometry using Topcon Auto-KR (Tomey Corporation, Nagoya, Japan) was 44.25/44.00@55 and 44.25/44.00@168 in the right and the left eyes, respectively. Despite the difference in visual acuity between the two eyes, the unilateral and alternate cover test was performed to rule out the possibility of esodeviation secondary to the accommodative spasm. No deviation was present at the two distances.

At the initial examination after the trauma, ACD in the right and left eyes were 3.08 mm and 1.87 mm, respectively. The calculated crystalline lens rise (CLR) at this time was 73 *μ*m in the right eye and 910 *μ*m in the left eye (Figure 1). Comparison of the AS-OCTs showed the anterior rotation of the ciliary body and the anterior part of the choroid in the left eye and ciliochoroidal effusion.

The baseline wavefront analysis using iTrace aberrometer (Tracey Technologies, Houston, TX) showed a significant amount of total aberration in the internal optics in the left eye compared to the right eye (0.245 *μ*m vs. 0.081 *μ*m) secondary to the increased amounts of total low- and high-order aberrations (Table 1). The positional changes secondary to trauma produced considerable changes in the internal optical aberrations of the left eye mostly crystalline lens so that there is a noticeable difference in the magnitude of low-order astigmatism (0.198 *μ*m vs. 0.036 *μ*m) and coma (0.110 *μ*m vs. 0.035 *μ*m) compared to the right eye in the initial assessment.

Cycloplegic refraction was evaluated with one drop of tropicamide 1% which was instilled every five minutes for three times, and auto refraction was repeated 30 minutes after the last drop.

The patient was reevaluated two weeks later. There were no changes in the right eye while the left eye showed significant improvement in the visual acuity, refractive error, and ACD. The uncorrected distance visual acuity was 1.0 with a refraction +1.00/−0.25 × 60° in the left eye. Keratometry readings did not show changes compared to the initial assessment, and IOP increased to 13.3 mmHg in the left eye.

**Figure 2:**
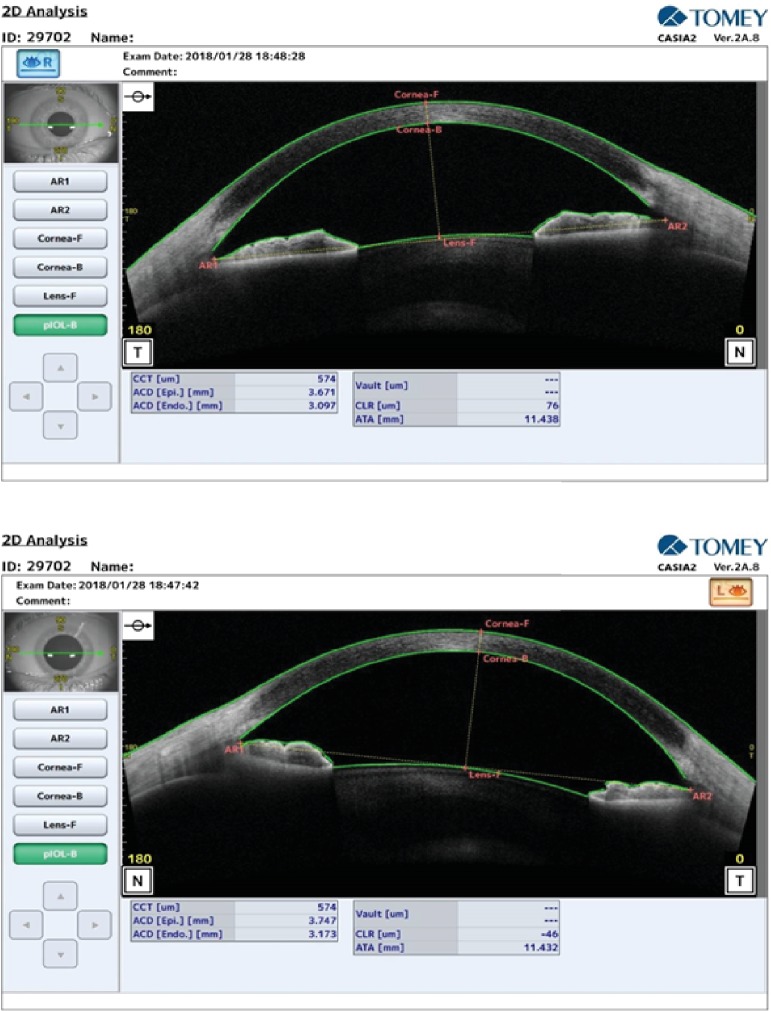
Anterior segment OCT two weeks after the trauma in the right and left eyes.

The left ACD increased to 1.30 mm and reached to 3.17 mm (vs. 3.09 in the right eye) while the CLR reduced by 956 *μ*m and obtained a negative value -46 *μ*m (Figure 2).

The next wavefront analysis at the two weeks posttrauma appointment showed a reduction of 0.187 *μ*m in the total internal optical aberrations. The lower- and higher-orders aberrations of the internal optics reduced by 0.149 *μ*m and 0.114 *μ*m, respectively. Associated with these improvements, root mean square of the internal lower-order astigmatism and coma changed significantly (Table 1).

## 3. Discussion

In this case, the ocular nonpenetrating trauma provided a myopic shift of about 7.00 D, which resolved after 15 days. This change was associated with the narrowing of ACD and increase in the CLR in the left eye. Comparison of Figures 1 and 2 in the left eye refers to the forward displacement of the iris-crystalline lens diaphragm secondary to ciliary body anterior rotation and ciliochoroidal effusion. It has been suggested that trauma in this area can increase the permeability of the blood vessels due to the involvement of the sympathetic nerve terminating in the walls of vessels [9]. Ultrasound biomicroscopy (UBM) is the best method to display the structures behind the iris and to show the ciliary rotation and choroidal effusion [8]; however, the obtained AS-OCT images partly highlighted these changes. Figure 1 visualizes the fluid in the suprachoroidal space in the left eye. In the obtained AS-OCT cut, there was not a continuous channel between the anterior chamber and the suprachoroidal space. Despite this, there was a reduced intraocular pressure in the left eye compared to the right eye in the initial assessment (7.9 mmHg vs. 13.6 mmHg). This difference in intraocular pressures may be attributed to the posttraumatic structural alternations.

Accommodative spasm has been mentioned as an etiologic factor of pseudomyopia after trauma [1, 10]; however, lack of difference in the manifest and cycloplegic refractions in the initial assessment and no eso-shift in the cover test ruled out the ciliary spasm as the cause of the induced myopia.

In conclusion, the traumatic myopia that develops after ocular blunt trauma is mostly a transient entity and caused by a combination of different factors such as ciliochoroidal effusion and forward shift of the lens-iris diaphragm. However, the precise contribution of each of the possible causes is difficult and can have individual variations from person to person. Anterior segment optical coherence tomography (AS-OCT) provides a precise method to better illustrate the anterior segment changes following blunt trauma.

## Figures and Tables

**Table 1:** Initial wavefront analysis and two weeks later.

WF summary		Initial Ass.						2 weeks later Ass.					
		Entire eye		Internal optics		Cornea		Entire eye		Internal optics		Cornea	
		OD	OS	OD	OS	OD	OS	OD	OS	OD	OS	OD	OS
Total		0.112	0.196	0.081	0.245	0.104	0.115	0.179	0.061	0.202	0.058	0.070	0.026

LOA	Total	0.098	0.137	0.036	0.198	0.090	0.106	0.172	0.045	0.188	0.049	0.055	0.005
	Defocus	0.0	0.0	0.0	0.0	0.0	0.0	0.0	0.0	0.0	0.0	0.0	0.0
	Astigmatism × axis°	0.098 × 179°	0.137 × 40°	0.036 × 32°	0.198 × 25°	0.090 × 168°	0.106 × 94°	0.172 × 1°	0.045 × 143°	0.188 × 9°	0.049 × 142°	0.055 × 132°	0.005 × 38°

HOA	Total	0.055	0.140	0.073	0.144	0.052	0.045	0.050	0.042	0.074	0.030	0.043	0.026
	Coma × axis°	0.010 × 4°	0.115 × 321°	0.035 × 177°	0.110 × 306°	0.045 × 359°	0.030 × 34°	0.041 × 58°	0.039 × 234°	0.060 × 97°	0.024 × 224°	0.038 × 319°	0.016 × 249°
	Trefoil × axis°	0.049 × 39°	0.047 × 71°	0.060 × 44°	0.065 × 62°	0.016 × 118°	0.032 × 107°	0.016 × 44°	0.010 × 115°	0.031 × 40°	0.008 × 81°	0.016 × 96°	0.014 × 6°
	Spherical	0.005	-0.014	-0.013	-0.023	0.018	0.009	0.001	-0.001	-0.011	-0.014	0.012	0.013

WF: wavefront; Ass.: assessment; LOA: lower-order aberration; HOA: higher-order aberration; OD: right eye; OS: left eye.

## References

[1] Ikeda N., Ikeda T., Kohno T. (2016). Traumatic myopia secondary to ciliary spasm after blunt eye trauma and reconsideration of its pathogenesis. *Graefe's Archive for Clinical and Experimental Ophthalmology*.

[2] Ikeda N., Ikeda T., Nagata M., Mimura O. (2002). Pathogenesis of transient high myopia after blunt eye trauma. *Ophthalmology*.

[3] Kim S. I., Cha Y. J., Park S. E. (2008). A case report on the change of the refractive power after a blunt trauma. *Korean Journal of Ophthalmology*.

[4] Rewri P., Goyal G., Ali W., Sharma A., Sood D. (2015). Acute onset bilateral myopia in convalescence phase of varicella infection. *Oman Journal of Ophthalmology*.

[5] Abraham L. M., Keembiyage R. D., Selva D., Casson R. (2006). Persistent unilateral myopia following blunt trauma. *Eye*.

[6] Doğanay S., Er H., Hepsen Í. F., Evereklioğlu C. (2001). Bilateral myopia following blunt trauma to one eye. *European Journal of Ophthalmology*.

[7] Kuchle M., Naumann G. O. (2003). Transient myopia after trauma. *Ophthalmology*.

[8] Lee J. W., Kwon S. J., Chai S. H., Kim H. K. (2009). A case of transient myopia after blunt eye trauma. *Japanese Journal of Ophthalmology*.

[9] Steele C. A., Tullo A. B., Marsh I. B., Storey J. K. (1987). Traumatic myopia; an ultrasonographic and clinical study. *The British Journal of Ophthalmology*.

[10] Chan R. V., Trobe J. D. (2002). Spasm of accommodation associated with closed head trauma. *Journal of Neuro-Ophthalmology*.

